# Clinicians’ Perspectives and Proposed Solutions to Improve Contraceptive Counseling in the United States: Qualitative Semistructured Interview Study With Clinicians From the Society of Family Planning

**DOI:** 10.2196/47298

**Published:** 2023-08-21

**Authors:** Rose Goueth, Kelsey Holt, Karen B Eden, Aubri Hoffman

**Affiliations:** 1 Department of Medical Informatics and Clinical Epidemiology Oregon Health & Science University Portland, OR United States; 2 Department of Family Community Medicine University of California, San Francisco San Francisco, CA United States; 3 Pacific Northwest Evidence-based Practice Center Department of Medical Informatics and Clinical Epidemiology Oregon Health & Science University Portland, OR United States; 4 Value Institute for Health & Care Dell Medical School The University of Texas at Austin Austin, TX United States

**Keywords:** contraceptive counseling, qualitative study, decision making, decision aids, clinician engagement, user-centered design, contraceptive, birth control, clinicians’ perspectives, patient-centered counseling, sexual health, family planning

## Abstract

**Background:**

Contraceptive care is a key element of reproductive health, yet only 12%-30% of women report being able to access and receive the information they need to make these complex, personal health care decisions. Current guidelines recommend implementing shared decision-making approaches; and tools such as patient decision aid (PtDA) applications have been proposed to improve patients’ access to information, contraceptive knowledge, decisional conflict, and engagement in decision-making and contraception use. To inform the design of meaningful, effective, elegant, and feasible PtDA applications, studies are needed of all users’ current experiences, needs, and barriers. While multiple studies have explored patients’ experiences, needs, and barriers, little is known about clinicians’ experiences, perspectives, and barriers to delivering contraceptive counseling.

**Objective:**

This study focused on assessing clinicians’ experiences, including their perspectives of patients’ needs and barriers. It also explored clinicians’ suggestions for improving contraceptive counseling and the feasibility of a contraceptive PtDA.

**Methods:**

Following the decisional needs assessment approach, we conducted semistructured interviews with clinicians recruited from the Society of Family Planning. The Ottawa Decision Support Framework informed the interview guide and initial codebook, with a specific focus on decision support and decisional needs as key elements that should be assessed from the clinicians’ perspective. An inductive content approach was used to analyze data and identify primary themes and suggestions for improvement.

**Results:**

Fifteen clinicians (12 medical doctors and 3 nurse practitioners) participated, with an average of 19 years of experience in multiple regions of the United States. Analyses identified 3 primary barriers to the provision of quality contraceptive counseling: gaps in patients’ underlying sexual health knowledge, biases that impede decision-making, and time constraints. All clinicians supported the development of contraceptive PtDAs as a feasible solution to these main barriers. Multiple suggestions for improvement were provided, including clinician- and system-level training, tools, and changes that could support successful implementation.

**Conclusions:**

Clinicians and developers interested in improving contraceptive counseling and decision-making may wish to incorporate approaches that assess and address upstream factors, such as sexual health knowledge and existing heuristics and biases. Clinical leaders and administrators may also wish to prioritize solutions that improve equity and accessibility, including PtDAs designed to provide education and support in advance of the time-constrained consultations, and strategic training opportunities that support cultural awareness and shared decision-making skills. Future studies can then explore whether well-designed, user-centered shared decision-making programs lead to successful and sustainable uptake and improve patients’ reproductive health contraceptive decision-making.

## Introduction

Over 200,000,000 people in the United States are of reproductive age, and over 90% of women report that they chose to use contraception [1]. Yet in the 2022 Kaiser Family Foundation Women’s Health Survey, only 30% of women reported that they had the information they needed to make decisions about contraception [1]. Women from Black, Hispanic, and Asian or Pacific Islander communities reported even lower access to information (28%, 26%, and 12%, respectively) [1]. While many studies focus on cost as the cause of disparities in contraceptive care, access to information remains a persistent barrier [2-4]. Even with federally mandated coverage, as few as 41% of women are aware that their insurance covers contraception [1]. Furthermore, access to information is essential to supporting men and women in making informed personal decisions about their reproductive health.

In 2022, The American College of Obstetricians and Gynecologists [5] updated their guidelines to state that the following:

Obstetrician-gynecologists (ob-gyns) should intentionally incorporate the reproductive justice framework into contraceptive counseling by ... prioritizing patients’ values, preferences, and lived experiences in the selection or discontinuation of a contraceptive method. Ob-gyns should adhere to the recommended ethical approach of shared decision making through patient-centered contraceptive counseling.

Shared decision-making is a process by which clinicians and patients share evidence (medical and personal), clarify which factors are most important to the patient in this decision (their “decision-making values”), and consider which option best achieves their top values in order to identify an informed, values-based treatment preference [6]. Decision-making is appropriate when considering 2 or more medically-relevant options, including decisions about changing or discontinuing therapy. Notably, shared decision-making also provides a systematic method for assessing patients’ knowledge, previous experiences, barriers, and needs—and to address any gaps—to ensure decisions are well-informed and feasible.

In situations in which there may be low access, knowledge, or consultation time, tools such as patient decision aids (PtDAs) can be provided before, during, or after the consultation. The National Quality Forum provides a list of key criteria for PtDAs [7], including that they: (1) provide high-quality, up-to-date medical evidence and a neutral, balanced, plain-language presentation of all relevant options; (2) identify that there are multiple options available, and that patients can be involved (as little or as much as they would like to be) in making a decision about which option might work best for their health goals and lifestyle; and (3) provide evidence-based decision support, including ways for patients to clarify—implicitly or explicitly—what matters most to them in this decision and to consider which options they prefer.

PtDAs can provide a quick, low-touch tool to help tailor clinical counseling (eg, by identifying gaps in knowledge or mismatches between values and preferences) and to systematically collect patient-reported preferences and outcomes [8]. Notably, PtDAs are often designed to address patients’ decisional conflict (uncertainty or anxiety that prevents taking action) in order to support behavior change and bridge the gap between thinking about and doing something to improve one’s health [6]. Since 1999, 8 Cochrane Collaboration reviews (105 randomized controlled trials with 31,043 participants worldwide) show that patients who use PtDAs have improved knowledge, more realistic expectations, less decisional conflict, and participate more actively in making decisions [8,9]. Several studies have shown that PtDAs improve engagement with, and continuation of, therapy, which is a long-term goal of this program of research in contraceptive counseling and reproductive health [8].

**Figure 1:**
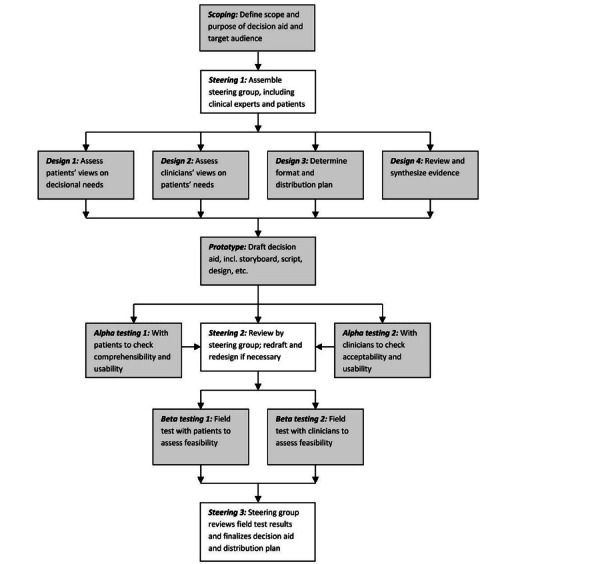
Model development process for decision aids (reproduced from Hoffman et al [12], which is published under Creative Commons Attribution 4.0 International License [13]).

In 2013, the International PtDA Standards collaboration summarized over 40 years of multidisciplinary research to describe the systematic design and delivery of high-quality PtDAs [10-12]. Figure 1 illustrates the PtDA Development Process Model, which is oriented in the Ottawa Decision Support Framework (ODSF).

It combines user-centered design, health communication, and mixed methods approaches to clearly understand patients’ and clinicians’ current experiences and needs, and to engage them in co-designing meaningful, feasible, accessible, and usable solutions.

The long-term goal of this program of research is to design an interactive contraceptive PtDA application, Healthy Sex Choices, and to test whether it improves patients’ knowledge, decision-making, engagement, and health outcomes. We have previously determined the scope of the tool, gathered an interprofessional steering committee, reviewed the literature, and conducted qualitative studies of patients’ experiences, needs, and design and access preferences. Therefore, the objective of this study was to assess clinicians’ experiences, including their perspectives on patients’ needs and barriers. We also explored clinicians’ suggestions for improving contraceptive counseling and the feasibility of a contraceptive PtDA.

## Methods

### Approach

When creating a user-centered PtDA, we want to understand current clinical practice from the perspective of patients and clinicians to improve and optimize these processes. International Patient Decision Aid Standard outlines specific steps (designs 1-4) within the Development Process Model (Figure 1) that will provide data for decision aid development and refinement. This study focused on Design 2 and Design 3 steps which emphasize the importance of understanding all potential users’ current experiences, barriers, needs, and preferences along with identifying the format in which these needs and preferences should be addressed. Interviews followed the approach described in MJ Jacobsen’s Decisional Needs Assessment in Populations workbook, which operationalizes the underlying theories of the ODSF [10,14]. The multidisciplinary research team included family planning doctors and nurse practitioners, decision scientists, informaticians, women’s health researchers, and designers.

### Ethics Approval

All study activities were reviewed and approved by Oregon Health and Science University’s (OHSU) institutional review board (#00022943). Participants who completed an interview and completed all advisory board tasks were provided with a US $35 gift card. The privacy and confidentiality of the deidentified data used for this research are currently preserved on an encrypted OHSU-owned hard drive within OHSU-firewalled cloud storage.

### Sample and Recruitment

We recruited clinicians who had experience conducting contraceptive counseling using purposive sampling and invitations posted to the Society of Family Planning’s (a multidisciplinary research community of all engaged in the science and medicine of abortion and contraception) community boards. All participants provided initial and ongoing consent, including signing e-consent forms via REDCap (Research Electronic Data Capture) before their interview date. Recruitment and interviews continued reaching thematic saturation (when analysis of data revealed no new themes) [15]. Five out of 15 clinicians were initially recruited to establish a clinician advisory panel for a larger PtDA feasibility study.

### Data Collection

Following the Decisional Needs Assessment Approach, the interview guide included 22 open-ended questions based on the underlying ODSF [16]. This framework identifies support needs for patients and clinicians dealing with complex decisions using 3 factors: decisional needs, decision support, and decisional outcomes [17]. Questions explored clinicians’ perspectives on patients’ current contraceptive decision-making process and needs; important components of contraceptive counseling; and the current provision of sexual health and contraceptive knowledge to patients. They also explored clinicians’ suggestions for improving contraceptive counseling for all patients. Interview questions were reviewed by an author (RG) and a group of clinical and decision-making experts, then tested by the multidisciplinary team to confirm concepts and optimize wording and flow.

A trained qualitative interviewer conducted interviews between December 2021 and February 2022, which were recorded and transcribed using WebEx’s videoconferencing software. The interviewer took field notes during all interviews, and reviewed, formatted, and checked all transcripts for accuracy.

### Data Analysis

The author (RG) uploaded and coded data in QDA Miner Lite (Provalis Research) [18]. Using an inductive iterative content analysis approach, initial codes were formed from the first 5 interviews [19,20]. Final themes were constructed with groupings from the final codebook. Major and minor themes were identified by frequency of the theme’s appearance. A theme qualified as major if its contents appeared in at least 7 of the 15 (50%) of the interviews. Additional codes were formed while deriving themes from interview data and all interviews were recoded with the finalized codebook.

## Results

### Participant Characteristics

Among the 15 participants, 12 were medical doctors (MD) and 3 were nurse practitioners (Table 1). Fourteen participants were female and had an average of 19 years of clinical experience (minimum 5.5, maximum 52 years). Ten currently practice in the Northeast, 3 practice in the West, and 2 practice in the South. Participants also work in a variety of clinical settings with community family planning and general health centers (7) and academic health centers (4) being the most prevalent. Interviews times ranged from 30 to 60 minutes, averaging 40 minutes.

**Table 1:** Clinician participant characteristics.

Characteristics		Values
**Clinician type, n (%)**		
	Medical doctor	12 (80)
	Nurse practitioner	3 (20)
**Sex, n (%)**		
	Female	14 (93)
	Male	1 (7)
Clinical experience (years), mean (range)		19 (5.5-52)
**Current practice location, n (%)**		
	Northeast	10 (67)
	Midwest	0 (0)
	South	2 (13)
	West	3 (20)

### Strengths and Challenges to Patient-Centered Counseling

When asked about the strengths of contraceptive counseling, a major theme was the acceptance of patient-centered care and shared decision-making models and pleasure with the shift toward models that stress reproductive autonomy and justice (Textbox 1). Though clinicians recognized the shift towards prioritization of autonomy, they identified 3 major challenges to providing patient-centered counseling: clinician and patient biases, time, and patient gaps in sexual health knowledge.

**Subtheme and relevant quote for primary theme challenges to patient-centered counseling.**

**Subtheme 1: Acceptance and use of patient-centered care**

- “I think that people counsel according to their training and understanding, and I think we’ve gotten much much better at understanding the process and the goal of patient-centered counseling and reproductive justice.” [17-year Medical Doctor, Northeast]

### Clinician and Patient Bias

We identified 3 subthemes related to clinician and patient bias (Textbox 2). Clinicians identified several forms of individual bias that can affect the patient-provider relationship and patients’ ability to exercise autonomy in decision-making. First, clinicians identified a major theme with the inherent biases within what they referred to as “LARC-first” (long-acting reversible contraception–first) counseling, where intrauterine devices and implants are emphasized as top-line methods for their superior effectiveness. This approach inherently steers women toward particular methods and is increasingly recognized as being a coercive practice. A minor theme was recognized by some; how biases patients hold (eg, racial or ethnic biases, suspicion of clinicians due to historical harms perpetuated by the health care system, etc) can affect their trust in the provider and patient-provider communication, a key to shared decision making. Last, clinicians acknowledged a major theme; their own racial or ethnic biases and how they can conflict with the care they provide, and the fact that they feel there are no useful tools to mitigate its presence in their work.

**Subthemes and relevant quotes for primary themes under challenges to patient-centered counseling.**

**Primary theme: clinicians and patient bias**

**Subtheme 1: issues with long-acting reversible contraception–first counseling**

- “I think that as providers, we always think that the effectiveness right is the most important thing and that’s not always that’s not anywhere close to true.” [17-year Nurse Practitioner, Northeast]

**Subtheme 2: patient biases affect provider trust and communication**

- “When I come across as a White older woman, people may not I think usually feel okay at sharing things with me, but they may kind of come in with a bias saying she’s just a White woman, what does she know? And then I have to kind of break down some of that to get into these areas that I know (a patient) might have some concerns about.” [17-year Nurse Practitioner, Northeast]

**Subtheme 3: effects of racial or ethnic biases**

- “Sometimes I ponder my ability to have shared decision-making conversations with people when we don’t share the same background and when there’s a lot of potential mistrust with the health care system. And ways in which we can try to either ameliorate or kind of get out of place where we can have that shared decision-making conversation.” [7-year Medical Doctor (MD), West]

**Primary theme: constraints of time**

**Subtheme 1: long patient interaction time**

- “I think it’s just hard to find that happy medium where you can, in an ideal world, we would all have an unlimited time to sit down with our patients and kind of dwindle it down to what they want. But you have to really strike a balance between providing them with enough information to make an informed decision without overwhelming them with too many options.” [17-year MD, South]

**Primary Theme: gaps in sexual health knowledge**

**Subtheme 1: low levels of sexual health knowledge barrier to decision-making**

- “Patients who have a first need for contraception, do not have adequate knowledge of the different methods available, or the mechanisms of the methods, or the different implications in terms of use or side effects.” [15-year MD, West]

**Subtheme 2: amount of knowledge correlated to the availability of sexual education**

- “I think in (Southern state), which provides has no kind of standard for sex ed in schools that my patients probably have less knowledge than I kind of experienced, witnessing it as a medical student in (Midwest state).” [6-year MD, South]

**Subtheme 3: misconceptions are rooted in knowledge deficits**

- “... a lack of basic knowledge about a woman’s body is doing a detriment to (patients).” [6-year MD, West]

### Constraints of Time

A major theme clinicians specified long patient interaction time as the greatest weakness of patient-centered counseling methods (Textbox 2). Several clinicians stated in high patient frequency environments, it is not always possible to assess patients’ priorities and values in a short counseling visit when patients need to leave with a contraceptive choice. They shared that some of their patients do not get to the decision-making portion of their visit due to the time required to educate or dispel misconceptions about sexual health.

### Gaps in Sexual Health Knowledge

Participants identified 3 subthemes related to gaps in sexual health knowledge (Textbox 2). A major theme clinicians identified was patients’ low level of sexual health knowledge being a barrier in guiding decision support. Some clinicians stated the amount can vary based on the patient’s personal experience, while others felt that patients generally do not have enough sexual health knowledge to make any informed decision. A minor theme arose when some clinicians correlated the amount of knowledge to the availability of comprehensive sexual education within the state. Common misconceptions addressed in clinical visits centered around reproductive anatomy (ie, pregnancy risk and irregular vs regular periods) and contraceptives’ mechanism of action (ie, frequency of adverse side effects and effects of hormones). Some misconceptions are rooted in knowledge deficits about sexual health, but many clinicians pointed to a lack of understanding of human anatomy’s interaction with contraceptives.

### Solutions to Improve Counseling

Clinicians reported various types of solutions to facilitate their ability to provide patient-centered counseling that would require a multilevel approach that addresses individual and systemic barriers. On the patient level, clinicians commonly identified 2 solutions: additional sexual health education provided to patients outside of counseling and deployment of decision-making tools to aid with the decision-making process. Clinicians thought these conversations could be delivered through other clinical staff trained to provide sexual health information. Most clinicians also saw value in employing decision-making tools for patients before a clinical visit to provide a foundation of knowledge for informed decision-making. Clinicians identified Bedsider as their number one and frequently referred option for decision-making support and sexual health information. This resource was consistently touted as a great place for patients to start their contraceptive decision-making process. Some participants who use Bedsider wished to see a decision-making tool attached to Bedsider’s current resources to offer a complete toolkit for patients to explore prior to their clinical visit.

At the clinician level, participants noted a minor theme: the importance of standardizing the teaching of contraceptive counseling for all involved in the contraceptive counseling process. Clinicians stated that experience and training can massively vary the kind of counseling a clinician performs. At the health center or system level, a common recommendation was to pass the responsibility of health education to clinical team members (ie, health educators and medical assistants) and increasing access to these members for overarching sexual health questions, potentially through telehealth visits. The most common sexual health topics clinicians suggested adding to current clinic sexual health education were consent, coercion and violence, and sexual satisfaction and wellness. Participants thought these topics may provide patients with the ability to further conversations about their overall sexual health. One clinician recommended scheduling subsequent appointments based on sexual health inquiries from patients to ensure all health needs are met.

Participants also brought up the need to improve health equity within contraceptive care. No specific solutions were provided but top suggestions all encompassed reproductive justice and patient-centered care values: meeting patients where they are at in their decision-making process, intentionally listening to patients, and empowering patient decision-making. Participants commonly identified a need to customize patient care to include identity complexity (ie, culture, disability, sexual orientation, and gender) and to not treat patients as a monolith:

Every contraceptive decision is like a clinical trial of one. So, I’m telling you what I think based on these population bases, like things of what we see, but, like, if it doesn’t work for you. Then we’ll change it.

42-year MD, Northeast

Clinicians acknowledged the increasing demand for contraceptive services, and a minor theme arose when they suggested implementing programs that reduce barriers to care (ie, provision in pharmacies, and contraceptive counseling via telehealth). Participants agreed they played a part in the solution to improving health equity but recognized that fixing problems within the overall US health care system would substantially improve the ability to provide equitable care.

Current structural hierarchies impede our ability to provide equitable care.

52-year MD, Northeast

## Discussion

### Principal Findings

Interviews with experienced family planning-focused providers revealed major barriers they face to providing patient-centered contraceptive counseling. Patient and clinician bias, patient encounter time, and gaps in patient sexual health knowledge were identified as contributors to subpar counseling, despite a general recognition that patient-centered counseling rather than directive counseling is now widely recognized as the standard of care.

To a certain extent, these barriers can be addressed at the individual level by using tools such as decision aids that support the patient-provider communication and counseling process. Decision aids can help in decreasing patient and clinician bias by providing a safe space for patients to explore treatment options that map to their preferences and values. The aids also provide the opportunity for patients to educate themselves on relevant health topics and treatment topics at their own pace, addressing the gaps in sexual health knowledge. Once the patient has a foundation of knowledge and potentially has preferred treatment options in mind (which is shared with the clinician prior to a visit), we optimize patient encounter time to shared decision-making for a treatment plan.

Our findings also suggest that complementing decision tools with system-level changes such as quality sex education and involvement of the clinical care team in contraceptive shared decision-making are also needed to comprehensively address barriers clinicians face in providing patient-centered counseling. Participants emphasized the need for addressing provider biases through standardizing contraceptive counseling and care and for extending opportunities for patients to develop sexual health knowledge in outlets other than the counseling encounter. Participants identified values that would aid in crafting viable health equity–centric solutions. Implementing these multilevel changes will require coordination and buy-in among several players (eg, policy makers, health care executives, etc) within reproductive health care.

Previous research echoes findings within our study. Biases and time constraints are known and persistent issues within health care that can erode patients’ overall health, decision-making, and care quality [21-23]. In the United States, sexual health education has become a polarizing topic, affecting the quality and accessibility of sexual health information within the education system [24,25]. Knowledge deficits can contribute to misconceptions about sexual health, such as perceived irregular menstrual patterns and human anatomy [26]. Clinicians try to review these topics within a clinical visit, but have a hard time providing a sufficient amount of information and supporting patient decision-making when time is the greatest limiting factor of current counseling processes [27].

This study’s unique contributions were directly asking providers about the challenges they face in conducting contraceptive counseling, mapping these challenges to a potential individual-level solution (PtDAs), and providing other clinician and health-system level solutions to improve counseling and care. Some solutions (given in the form of guidelines) suggested for improving counseling are well-researched and provide greater access to underserved populations. Patient decision aids and telehealth counseling visits have decreased health care costs, improved patient outcomes, and increased access to health care and information [28-31]. Implementing measures like the Person-Centered Contraceptive Counseling scale can also aid clinicians in evaluating the effectiveness of their contraceptive counseling methods [32]. These solutions can also be tailored to patients’ various identities, aiding in the removal of bias and widening access to reproductive health services.

### Limitations

Our study only included a small, focused sample of family planning-focused clinicians, not capturing the sentiments of all clinicians who support contraceptive counseling and care. Small sample sizes are common for research informing the development of decision aids [33]. Future research should capture the sentiments of a larger population of clinicians with a range of clinical backgrounds and patient populations to better understand the current climate of contraceptive counseling efforts. Our research mostly included clinicians who currently practice within the Northeast region. Many clinicians had training outside of the Northeast, but future research would greatly benefit from perspectives found outside of the Northeast, especially focusing on the Southern and Midwestern regions as many contraceptive and abortion care legislation and policies have transformed the way clinicians engage in contraceptive care [34].

### Conclusions

Clinicians who conduct contraceptive counseling recognize the complexity of current counseling practices and the need for improving counseling and care. Our findings demonstrate (1) how solutions like PtDAs can improve contraceptive counseling to acknowledge time constraints, address patient and clinician biases, and create a foundation of sexual health knowledge, and (2) viable considerations and solutions for improving access and quality of contraceptive counseling. The overall improvement of counseling and care should be crafted through a multilevel approach, including improved clinician training and outlets for addressing patient sexual health knowledge gaps at the forefront of these solutions.

## Abbreviations

LARC: long-acting reversible contraception
MD: medical doctor
ODSF: Ottawa Decision Support Framework
OHSU: Oregon Health and Science University
PtDA: patient decision aid
REDCap: Research Electronic Data Capture

## Data Availability

The data sets generated and analyzed during the study are not publicly available nor can be shared privately due to institutional review board protocol restrictions.
